# FoxO proteins or loss of functional p53 maintain stemness of glioblastoma stem cells and survival after ionizing radiation plus PI3K/mTOR inhibition

**DOI:** 10.18632/oncotarget.10702

**Published:** 2016-07-19

**Authors:** Elke Firat, Gabriele Niedermann

**Affiliations:** ^1^ Department of Radiation Oncology, University Hospital Freiburg, Freiburg, Germany; ^2^ German Cancer Consortium (DKTK), Freiburg, Germany; ^3^ German Cancer Research Center (DKFZ), Heidelberg, Germany

**Keywords:** cancer stem cells, radiotherapy, FOXO, p53, glioblastoma

## Abstract

Dual PI3K/mTOR inhibitors do not effectively radiosensitize glioblastoma multiforme stem cells (GBM-SCs), but p53-proficient GBM-SCs are more responsive than p53-deficient ones. Here, we found that p53-proficient, but not p53-deficient, GBM-SCs lost stemness and differentiated after γ-irradiation combined with PI3K/mTOR inhibition; expression of FoxO proteins was also lost. FoxO overexpression inhibited the loss of stem cell markers under these conditions. Combined, but not single, FoxO1/3 deletion or pharmacological inhibition of FoxO transcriptional activity strongly reduced stem and progenitor marker expression, particularly that of Sox2. Binding of FoxO1 and FoxO3 to the *sox2* regulatory regions was also found. However, combined FoxO1/3 knockdown strongly reduced self-renewal and post-treatment survival only in p53-proficient GBM-SCs. This suggests that FoxO1 and FoxO3 are crucial for functional stemness and post-treatment survival mainly in p53-proficient but not in p53-deficient GBM-SCs, and that these functions can be maintained through the loss of DNA damage-responsive p53 instead.

## INTRODUCTION

Stem-like tumor cells, commonly called cancer stem cells (CSCs), are undifferentiated tumor cells with high self-renewal and impaired differentiation capacity [1, 2]. Although there is some controversy on cell surface markers expressed by glioblastoma multiforme stem cells (GBM-SCs), there is nevertheless strong evidence for differentiation hierarchies in GBMs, with undifferentiated stem-like cells at the apex [3]. Xenotransplantation experiments using patient-derived stem-like cells [4–6] and syngeneic mouse glioma models [7] have shown that glioma formation depends on undifferentiated, stem-like but not on differentiated tumor cells. CSCs are often more resistant to genotoxic treatments than differentiated tumor cells, and thus are particularly important for resistance to conventional chemo- and radiotherapy [7–9].

We have studied inhibitors of the phosphatidylinositol 3-kinase (PI3K)/Akt/mammalian target of rapamycin (mTOR) pathway as potential radiosensitizers for GBM-SCs because this pathway is important for cell survival–particularly under stress including that induced by γ-irradiation (γIR); moreover, it coregulates stemness in normal stem cells and CSCs [10–15]. However, in a recent study, we surprisingly found that PI3K/Akt/mTOR inhibitors do not generally promote γIR-induced cell death. Whereas p53-deficient GBM-SCs showed reduced γIR-induced cell death when a dual PI3K/mTOR inhibitor was added, p53-proficient ones underwent slightly increased cell death [16].

Here, we further analyzed the effects of combination treatment on patient-derived GBM-SCs with different functional p53 status, and found fundamental differences in the expression of stemness markers and forkhead box O (FoxO) transcription factors (TFs). The TF p53 is a major tumor suppressor and has also been implicated in the regulation of stemness and differentiation [17–22]. FoxO TFs (comprising FoxO1, FoxO3a, FoxO4, and FoxO6 in mammals) are downstream of and inhibited by the PI3K/Akt/mTOR pathway. Functionally, they appear to be partially redundant [23, 24]. They share many target genes with p53 and co-regulate the genotoxic stress response [24–27]. However, both anti- and pro-tumoral effects have been ascribed to FoxO proteins [23, 24, 28–34]. Furthermore, FoxO proteins promote longevity [25] and are essential for stemness in mammalian embryonic stem (ES) cells [29], hematopoietic stem cells (HSCs) [35–37], neural stem cells (NSCs) [38–40], muscle stem cells [41], and leukemia stem cells [28, 32], as well as stem cells of the immortal *Hydra* [42].

Little is known about how FoxO proteins contribute to stemness in solid-tumor CSCs. Sunayama *et al.* reported that FoxO3a knockdown reduced the expression of differentiation markers in patient-derived GBM-SCs and that constitutively active FoxO3a induced GBM-SC differentiation [43]. This led them to conclude that FoxO3 is required for differentiation and inhibits stemness of GBM-SCs, apparently opposite to its role in normal NSCs [38–40]. In breast cancer stem-like cells, FoxO3 activation by Akt inhibition reduced stemness and triggered cell death; overexpression of dominant-negative FoxO3 retained stem cell marker expression and viability [44]. Similar findings were reported by others [45, 46]. In prostate cancer cells, FoxO3a knockdown increased the expression of the CSC markers CD133 and CD44 and also sphere formation, a surrogate marker for the self-renewal activity of CSCs [47]. Taken together, these studies so far suggest that FoxO3 inhibits stemness and survival of solid-tumor CSCs. There are as yet no reports on the role of FoxO1 in regulating stemness in solid tumors.

Here, in contrast, we provide evidence that FoxO proteins are crucial for maintaining stemness and cell survival in GBM-SCs, particularly those with functional p53. In addition, the data suggest that CSC stemness and survival can be maintained through the loss of DNA damage-responsive p53 instead.

## RESULTS

### Combined treatment with γIR and a dual PI3K/mTOR inhibitor causes loss of stemness and of FoxO proteins in p53-proficient GBM-SCs

The patient-derived GBM-SC lines used in this study (GBM4, 10, 22, 36, and G166 [48, 49]) displayed heterogeneous expression of PI3K/Akt/mTOR pathway components. The expression levels of the classical stem and progenitor cell markers Sox2, Musashi, and Nestin differed only slightly. All tested GBM-SC lines expressed FoxO1 and FoxO3a, but not FoxO4 (Figure S1A). There were also differences in the functionality of p53 as assessed after DNA-damaging γIR (GBM10, 22, and 36: functional p53; GBM4 and G166: non-functional p53) (Figure S1B).

**Figure 1:**
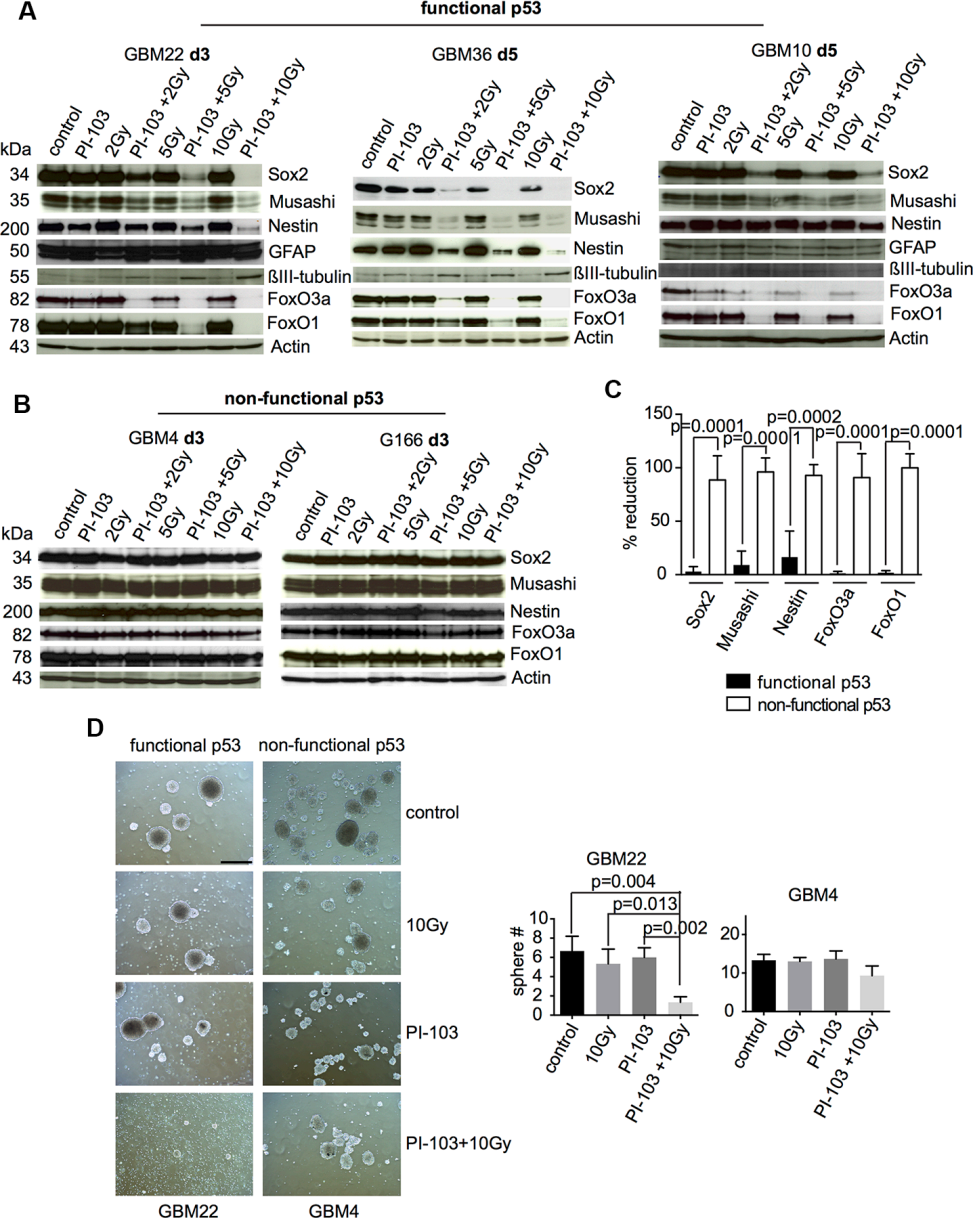
Combination treatment with γIR and PI-103 causes the loss of stemness markers and FoxO proteins in p53-proficient GBM-SCs p53-proficient GBM-SCs (**A**) or p53-deficient GBM-SCs (**B**) were incubated with 0.5 μM PI-103 for 1 h and then irradiated. Samples were analyzed by Western blot at the time points indicated. Actin served as loading control. Shown are representative results from 4 (GBM22), 2 (GBM36), 2 (GBM10), 3 (GBM4), and 2 (G166) experiments, respectively. (**C**) Quantification of changes in the expression of stem and progenitor markers and of FoxO proteins after combination treatment with 10 Gy + PI-103 compared to untreated cells; black columns: p53-proficient GBM-SCs; open columns: p53-deficient GBM-SCs. (**D**) Single cells with functional or non-functional p53 were treated for 1 h with 0.5 μM PI-103 and then irradiated. Three days later, the cells were analyzed for sphere formation. *n* = 3 experiments. The bar represents 500 μm.

Combination treatment with γIR and the dual PI3K/mTOR inhibitor PI-103, but not single treatments, caused the downregulation of stem and progenitor cell markers as well as of FoxO1 and FoxO3 in GBM-SCs with functional p53 (Figure 1A and 1C) but not in those with non-functional p53 (Figure 1B and 1C). Radiation doses of 2, 5, and 10 Gy were tested. A daily dose of 2 Gy is typically applied in conventional fractionated radiotherapy; doses of 5 and 10 Gy are applied in hypofractionated radiotherapy. The downregulation of the stem cell markers and of FoxO proteins in combination-treated, p53-proficient GBM-SCs was already found at 2 Gy, but was usually more pronounced at the higher radiation doses. Downregulation of these proteins and weak upregulation of the differentiation markers glial fibrillary acidic protein (GFAP) and βIII-tubulin in the p53-proficient GBM-SCs was usually found 3–5 days after the combination treatment (Figure 1A) and later (Figure S2A and S2C). The p53-proficient GBM10-SC line only transiently downregulated the stem and progenitor cell markers and FoxO proteins after 5 days. Loss of stem cell marker expression correlated with the loss of sphere formation (Figure 1D). As described previously [16], after the combination treatment, cell death was slightly increased in p53-proficient CSCs (Figures S2B, 2C, 3C, 4C, 5C, 6C) and reduced in p53-deficient ones (Figures S2D, 3F, 4F, 5H, 6G) compared to irradiation alone. Similar results were found when, instead of PI-103, the dual PI3K/mTOR inhibitor NVP-BEZ235 was combined with γIR (Figure S3). These results suggested that, in p53-proficient GBM-SCs, the combination treatment with γIR and a dual PI3K/mTOR inhibitor causes the loss of stemness and the induction of differentiation associated with a low amount of cell death. The results also suggested that the loss of expression of stem and progenitor markers and of FoxO proteins may depend on functional p53.

### p53 knockdown inhibits the loss of stemness and of FoxO proteins in GBM-SCs treated with γIR plus a PI3K/mTOR inhibitor

**Figure 2:**
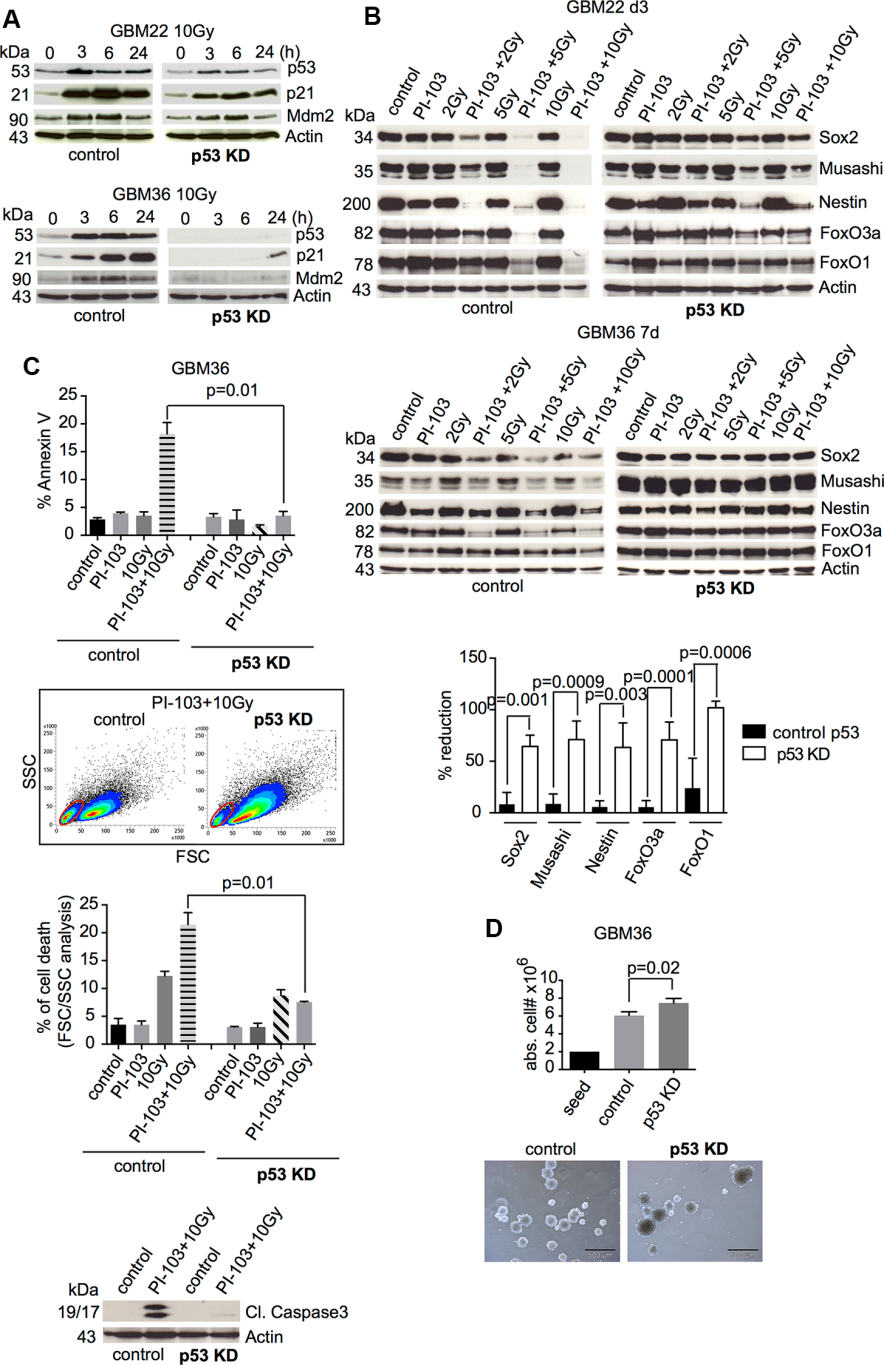
Knockdown of p53 blocks the loss of stemness markers and FoxO proteins after combination treatment with γIR and PI-103 (**A**) Assessment of shRNA knockdown efficiency by Western blot after 10-Gy γIR (shown are representative results from 3 experiments for each cell line; for statistical analysis, see Figure S4). (**B**) Control shRNA- or p53 shRNA-transduced GBM-SCs were incubated with PI-103 (1 μM) and irradiated 1 h later. Western blot analyses were performed after 3 or 7 days. Shown are representative results from 3 (GBM22) and 2 (GBM36) experiments, respectively. Quantification of changes in the expression of stem and progenitor markers and of FoxO proteins after combination treatment with 10 Gy + PI-103 compared to untreated cells; black columns: p53-proficient GBM-SCs; open columns: p53-knockdown cells. (**C**) Control or p53 knockdown GBM-SCs were treated with PI-103 (0.5 μM) for 1 h and irradiated with 10 Gy. Apoptosis was assessed at day 5 by flow-cytometric detection of annexin V staining, FSC/SSC analysis (*n* = 3 experiments), and Western blot for cleaved caspase 3 (1 of 2 experiments with similar results is shown). (**D**) Absolute cell numbers and sphere-forming capacity of control or p53 knockdown GBM-SCs after 3 days of incubation in CSC medium (*n* = 3 experiments). The bars represent 500 μm. Apoptosis and proliferation data in (C) and (D) represent means ± SD. KD, knockdown.

To prove whether combination treatment-induced loss of stemness markers and of FoxO proteins indeed depends on functional p53, we performed an shRNA-mediated p53 knockdown in two p53-proficient GBM-SC lines (GBM22 and GBM36). Inactivation of p53 was confirmed by lack of its upregulation and that of downstream targets after γIR (Figure 2A and Figure S4). As shown in Figure 2B, the p53 knockdown inhibited the loss of stemness markers and of FoxO proteins after γIR/PI-103 combination treatment. The slight apoptosis usually seen in combination-treated p53-proficient GBM-SCs was also reduced and correlated with decreased cleavage of caspase 3 (Figure 2C). Cell proliferation and sphere size were increased in p53 knockdown CSC cultures (Figure 2D). These results confirmed that the treatment-induced loss of stemness and of FoxO proteins depended on functional p53.

### FoxO3 knockdown slightly reduces stemness markers and in p53-proficient GBM-SCs strongly increases γIR- or γIR/PI-103-induced cell death

**Figure 3:**
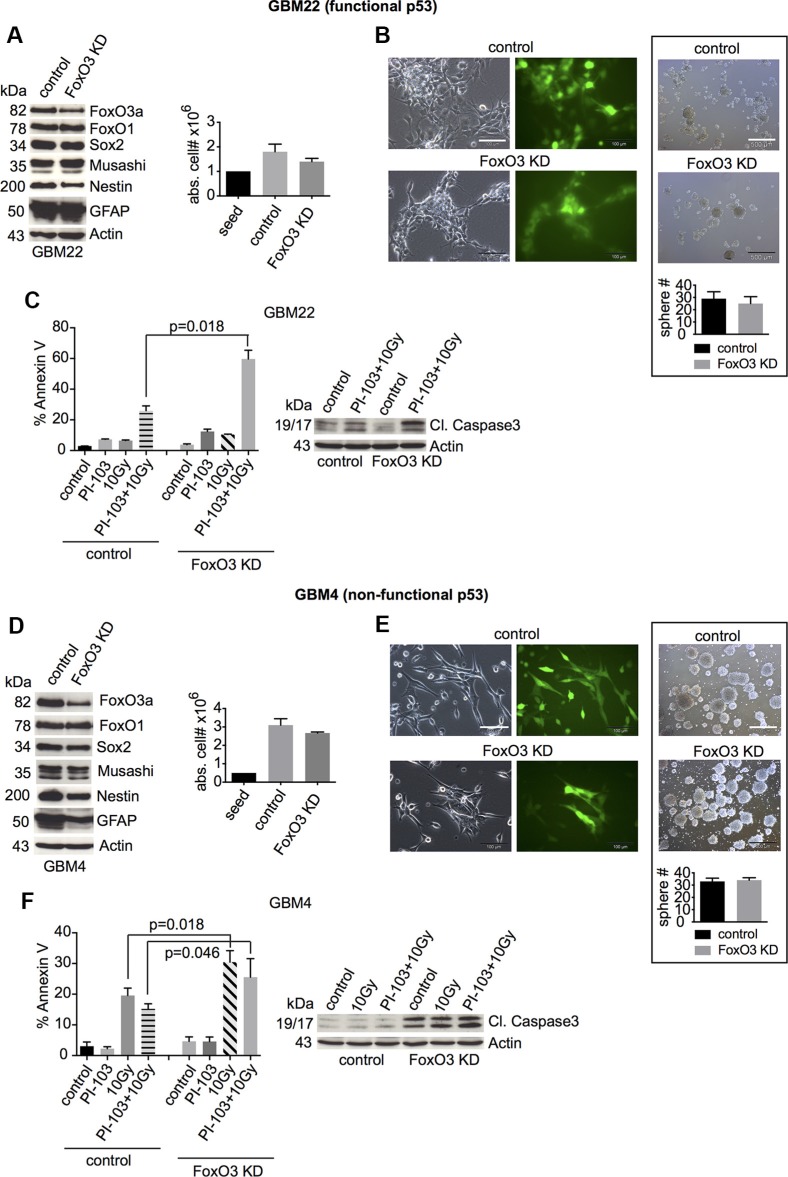
FoxO3 knockdown slightly reduces the expression of stemness markers and strongly increases cell death after γIR or combination treatment with γIR and PI-103 (**A** and **D**, left) Western blot analyses of the expression of FoxO proteins, stemness markers, and the differentiation marker GFAP in control shRNA- or FoxO3 shRNA-transduced GBM-SCs (*n* = 3 experiments; for statistical analysis, see Figure S4). (A and D, right) Absolute cell numbers after 3 or 4 days of incubation in CSC medium. (**B** and **E**, left) Confirmation of the transduction efficiency by GFP expression analysis. The bar represents 100 μm. (B and E, right) Sphere-forming capacity after 3 days of incubation in CSC medium. The bar represents 500 μm. (**C** and **F**) Assessment of cleaved caspase 3 (1 of 2 experiments is shown, each with similar results) and apoptosis by annexin V staining. Cells were treated with 0.5 μM PI-103 for 1 h and then irradiated with 10 Gy. The analyses were performed after 2 (GBM22) or 4 days (GBM4). Data for cell and sphere numbers and apoptosis in (A)–(F) represent means ± SD from 3 independent experiments. KD, knockdown.

FoxO3 seems particularly important in the nervous system [38, 39]. Therefore, we next assessed its influence on stemness and cell death of GBM-SCs. FoxO3 shRNA knockdown reduced the expression of Nestin and Sox2 slightly, but the reduction reached statistical significance only for Nestin in GBM22-SCs and for Sox2 in GBM4-SCs (Figure 3A and 3D, left and Figure S4). Similar to what has been reported by Sunayama *et al.* [43], expression of the differentiation marker GFAP was significantly reduced, but only in p53-deficient GBM-SCs (Figure 3D and Figure S4). Proliferation and sphere formation were not significantly decreased (Figure 3A and 3B, 3D and 3E). However, upon γIR or combination treatment with γIR and PI-103, cell death was increased, particularly in p53-proficient cells (Figure 3C, 3F, left, and S5A). This was accompanied by increased levels of cleaved caspase 3 (Figure 3C, 3F, right). Taken together, these results point to an anti-apoptotic effect of FoxO3 in GBM-SCs, particularly in p53-proficient ones.

### FoxO1 knockdown marginally reduces the expression of stemness markers and cell death after γIR or γIR/PI-103 combination treatment

**Figure 4:**
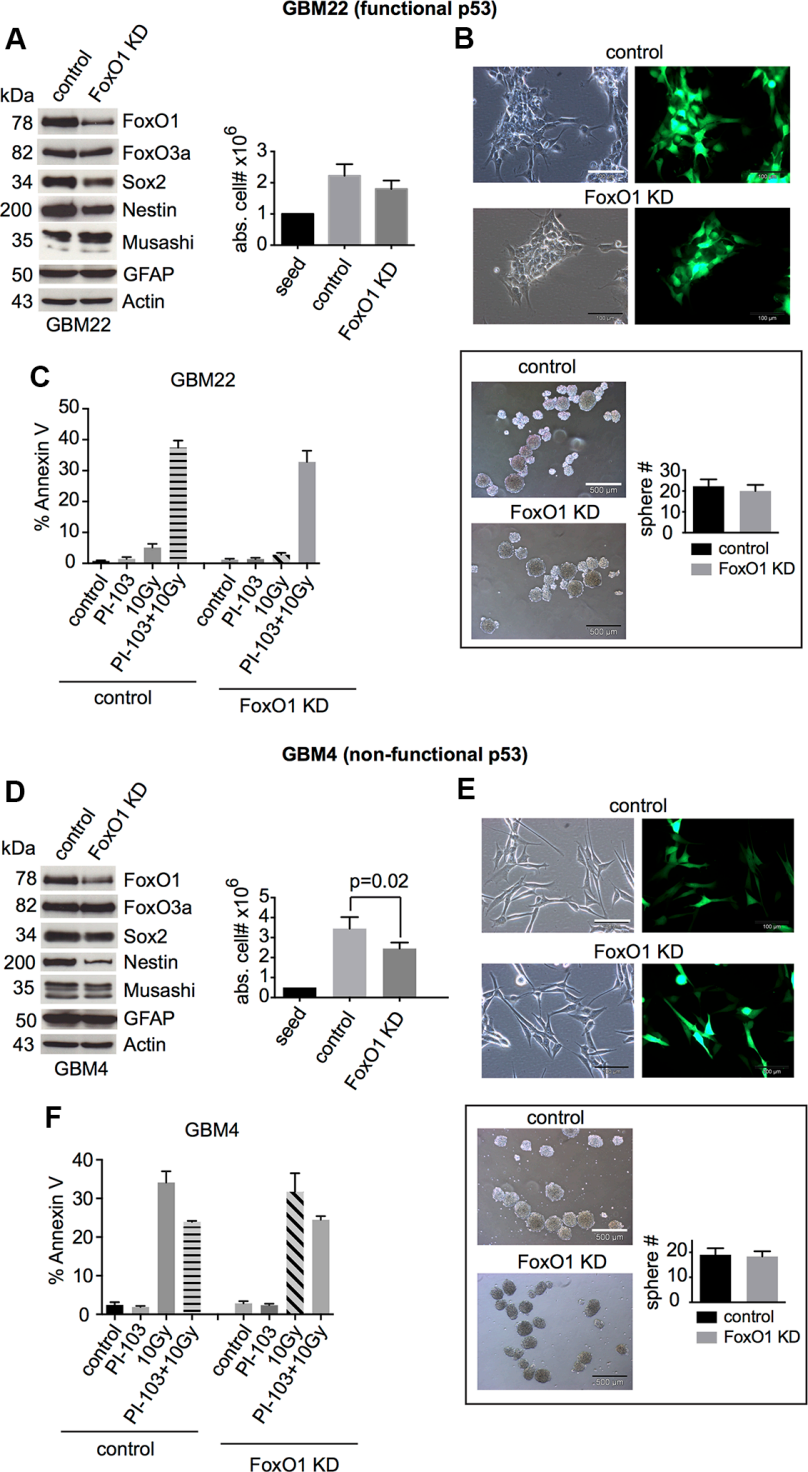
FoxO1 knockdown slightly reduces the expression of stemness markers and does not increase cell death after γIR or combination treatment with γIR and PI-103 (**A** and **D**, left) Western blot analyses of control shRNA- or FoxO1 shRNA-transduced GBM-SCs assessing the expression of FoxO proteins, stemness markers, and the differentiation marker GFAP (*n* = 3 experiments; for statistical analysis, see Figure S4). (A and D, right) Absolute cell numbers after 4 days of incubation in CSC medium. (**B** and **E**, upper) Confirmation of the transduction efficiency by GFP expression analysis. The bar represents 100 μm. (B and E, lower) Sphere-forming capacity after 3 days of incubation in CSC medium. The bar represents 500 μm. (**C** and **F**) Assessment of apoptosis by annexin V staining. Cells were treated with 0.5 μM PI-103 for 1 h and then irradiated with 10 Gy. The analyses were performed after 2 (GBM22) or 6 days (GBM4). Data for cell and sphere numbers and apoptosis in (A)–(F) represent means ± SD from 3 independent experiments. KD, knockdown.

FoxO1 is essential for pluripotency in ES cells [29] and for maintaining an undifferentiated state in normal NSCs [40]. In the GBM-SCs studied by us, FoxO1 shRNA knockdown only slightly reduced proliferation and expression of the stem and progenitor cell markers Sox2 and Nestin; statistical significance was only reached for GBM22 (Figures 4A, 4D and S4). Sphere formation was not significantly reduced (Figure 4B, 4E). Cell death (Figures 4C, 4F and S5B) was not increased upon γIR or combination treatment with γIR and PI-103. These results indicate that FoxO1 may play some role in the maintenance of stemness in GBM-SCs.

### Combined FoxO1/3a knockdown abolishes stem cell marker expression, but sphere formation and cell survival upon γIR/PI-103 treatment are mainly reduced in p53-proficient GBM-SCs

**Figure 5:**
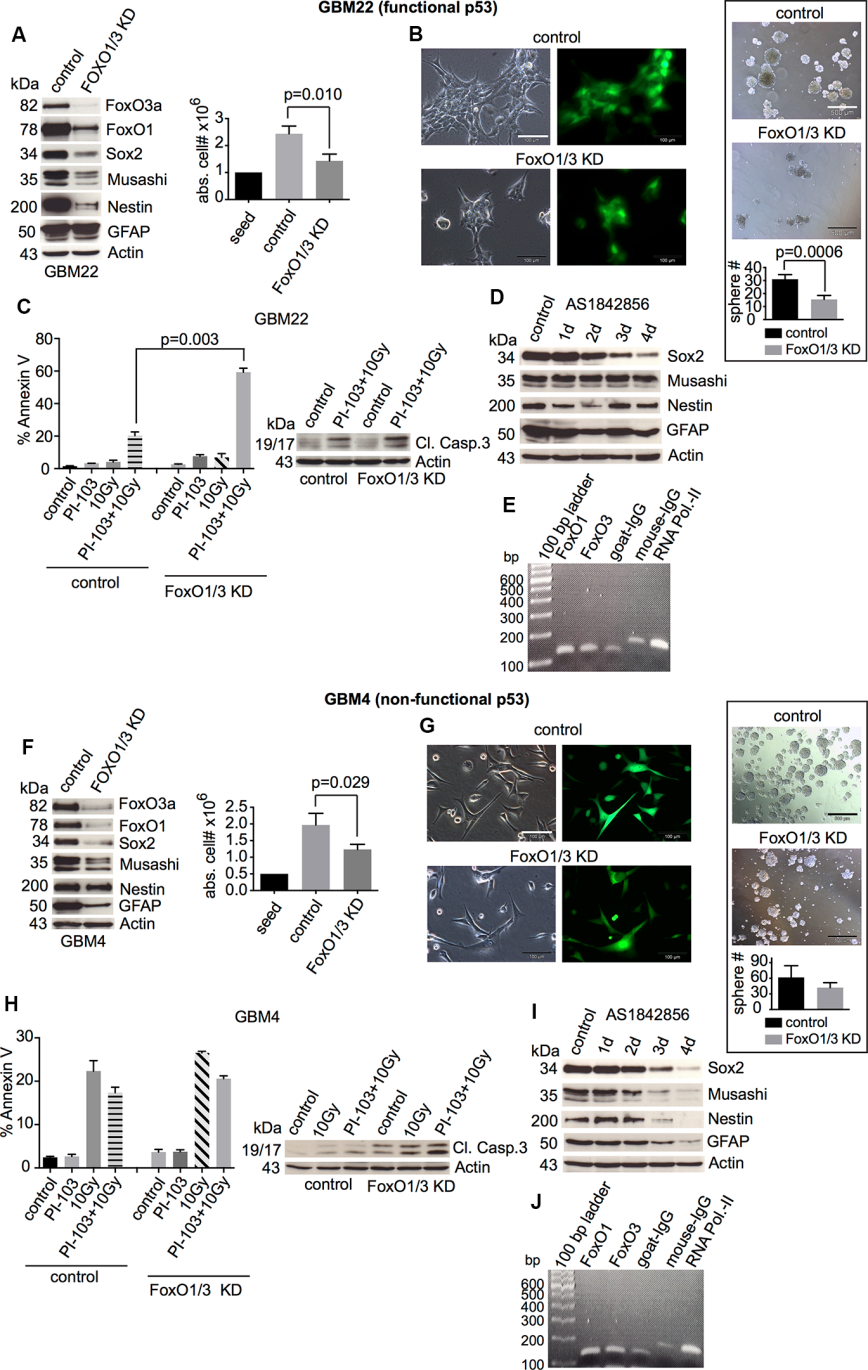
Effects of the combined FoxO1/3 knockdown on stemness and survival of GBM-SCs depending on p53 functionality (**A** and **F**, left) Western blot analyses of control shRNA- or FoxO1/3 shRNA-transduced GBM-SCs assessing the expression of FoxO proteins, stemness markers, and the differentiation marker GFAP (*n* = 3 experiments; for statistical analysis, see Figure S4). (A and F, right) Absolute cell numbers after 3 or 4 days of incubation in CSC medium. (**B** and **G**, left) Confirmation of the transduction efficiency by GFP expression analysis. The bar represents 100 μm. (B and G, right) Sphere-forming capacity after 3 days of incubation in CSC medium. The bar represents 500 μm. (**C** and **H**) Assessment of cleaved caspase 3 (1 of 2 experiments is shown, each with similar results) and of apoptosis by annexin V staining. Cells were treated with 0.5 μM PI-103 for 1 h and then irradiated with 10 Gy. The analyses were performed after 2 (GBM22) or 4 days (GBM4). (**D** and **I**) Western blot analyses of stemness markers and GFAP after incubation with 10 μM of the FoxO inhibitor AS1842856 (1 of 2 experiments is shown, each with similar results). (**E** and **J**) ChIP assay: DNA from GBM-SCs co-immunoprecipitated with anti-FoxO1, anti-FoxO3, or control antibody (goat IgG) was amplified by PCR using primers specific for the *sox2* regulatory regions. Precipitation with anti-RNA polymerase II and the respective mouse IgG control antibody was performed to validate the assay (1 of 2 experiments is shown, each with similar results). Data for cell and sphere numbers and apoptosis in (A)–(C) and (F)–(H) represent means ± SD from 3 independent experiments. KD, knockdown.

Combined FoxO1/3a knockdown strongly diminished stem and progenitor cell marker expression (Figures 5A, 5F, left and S4) and decreased cell proliferation, independent of the functional p53 status (Figure 5A, 5F, right). However, sphere formation was strongly reduced only in p53-proficient GBM-SCs (Figure 5B, 5G, right). Likewise, combination treatment-induced apoptotic cell death was strongly increased only in p53-proficient GBM-SCs, correlating with cleaved caspase 3 (Figures 5C, 5H and S5C). Using AS1842856, an inhibitor of FoxO1 and (to a lesser extent) FoxO3 transcriptional activity [50], we confirmed that the expression of stem and progenitor cell markers in GBM-SCs, particularly that of Sox2, depends on the transcriptional activity of FoxO proteins (Figure 5D and 5I).

FoxO1, but not FoxO3, binds to the *sox2* regulatory regions in ES cells [29, 51]. Since strong reduction in stem cell marker expression occurred only in FoxO1/3a double-deficient GBM-SCs (see Figures 5A, 5F, left, and S4) and FoxO3a appears more important than FoxO1 in normal NSCs [38, 39], we assessed the binding of both FoxO1 and FoxO3a to the *sox2* regulatory region in GBM-SCs. Chromatin immunoprecipitation (ChIP) indeed revealed binding of both FoxO1 and FoxO3a to the regulatory regions of the *sox2* gene in GBM-SCs, independent of the p53 status (Figure 5E and 5J). This, together with the strong reduction of stem cell marker expression only in FoxO1/3 double-knockdown cells, suggests that both FoxO proteins are required for the maintenance of stem cell marker expression in GBM-SCs. However, as indicated by the results presented above, FoxOs (and Sox2) may be required for functional stemness and post-treatment survival mainly in p53-proficient (see Figure 5B, 5C), but not in p53-deficient (see Figure 5G, 5H), GBM-SCs.

### FoxO3 overexpression inhibits the post-treatment downregulation of stem and progenitor markers

**Figure 6:**
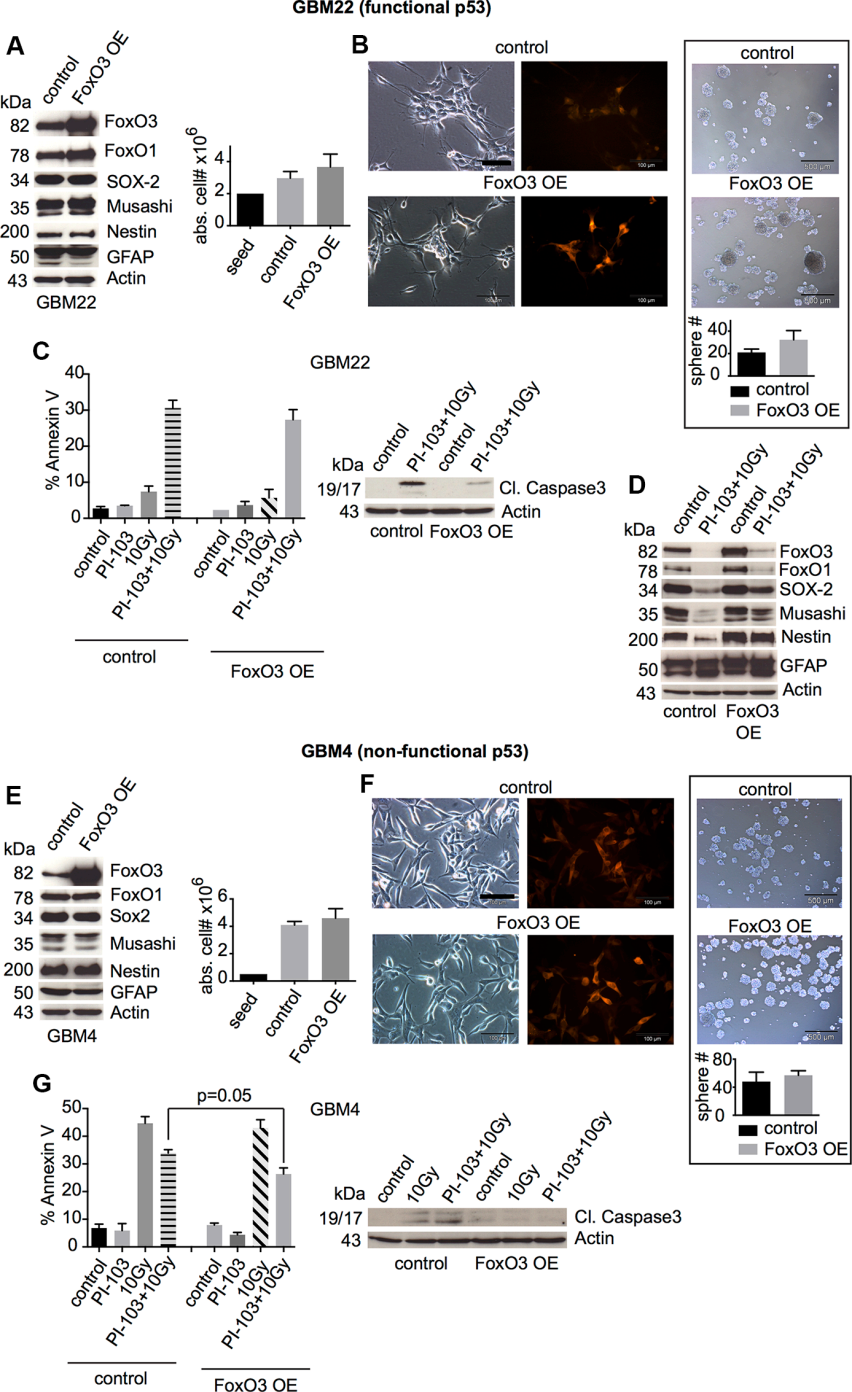
FoxO3 overexpression inhibits the downregulation of stemness markers after combination treatment with γIR and PI-103 in p53-proficient GBM-SCs (**A** and **E**, left) Western blot analysis of the expression of FoxO proteins, stem cell markers, and GFAP in GBM-SCs transduced with control or FoxO3-encoding lentiviral particles (*n* = 3 experiments; for statistical analysis, see Figure S4). (A and E, right) Absolute cell numbers after 2 or 4 days of incubation in CSC medium. (**B** and **F**, left) Confirmation of the transduction efficiency by RFP expression analysis. The bar represents 100 μm. (B and F, right) Sphere-forming capacity after 3 days of incubation in CSC medium. The bar represents 500 μm. (**C** and **G**) Assessment of cleaved caspase 3 (1 of 2 experiments is shown, each with similar results) and of apoptosis by annexin V staining. Cells were treated with 0.5 μM PI-103 for 1 h and then irradiated with 10 Gy. The analyses were performed after 2 (GBM22) or 6 days (GBM4). (**D**) Western blot analyses of control or FoxO3-overexpressing GBM22-SCs showing that FoxO3 overexpression inhibits the downregulation of stemness markers in p53-proficient GBM-SCs (1 of 2 experiments is shown, each with similar results). The cells were treated as indicated in (C) and (G) and analyzed after 2 days. Data for cell and sphere numbers and apoptosis in (A)–(C) and (E)–(G) represent means ± SD from 3 independent experiments. OE, overexpressing.

FoxO3 overexpression did neither influence the basal expression of stem and progenitor cell markers nor cellular proliferation or sphere formation (Figures 6A, 6B, 6E, 6F, and S4). γIR/PI-103-induced cell death was slightly reduced (for GBM4, the reduction almost reached statistical significance: *p* = 0.05), correlating with reduced levels of cleaved caspase 3 (Figures 6C, 6G, and S6). In p53-proficient GBM-SCs, FoxO3 overexpression inhibited the loss of stem and progenitor cell markers, which usually occurred upon γIR/PI-103 combination treatment in p53-proficient CSCs (Figure 6D; see also Figure 1A). Hence, these overexpression experiments confirmed the role of FoxO proteins in the maintenance of stemness and post-treatment survival of GBM-SCs, depending on the expression of DNA damage-responsive p53.

## DISCUSSION

**Figure 7:**
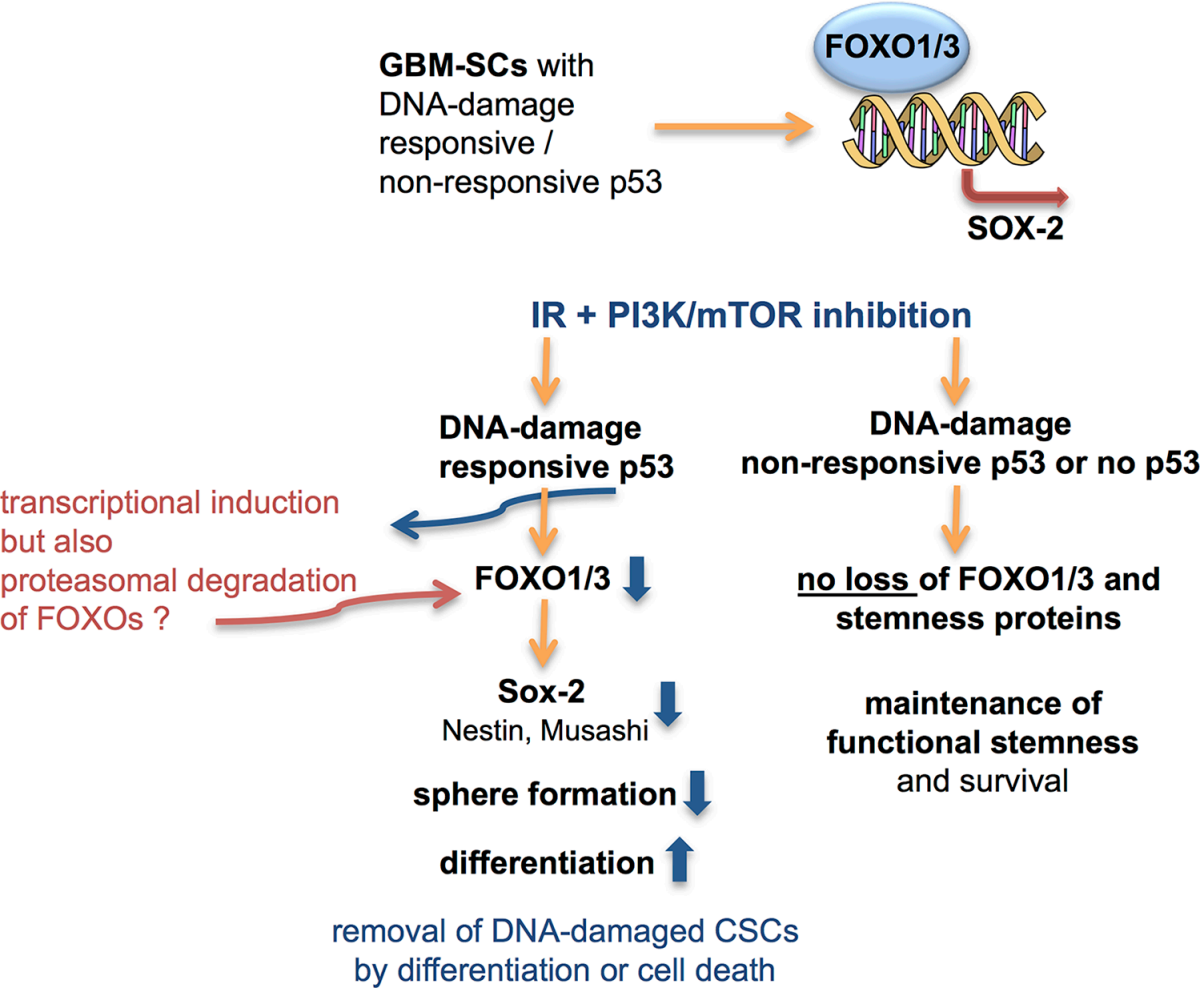
Proposed role of FoxOs and p53 in the control of stemness and post-treatment survival in GBM-SCs FoxO1/3a single and double knockdowns, pharmacological inhibition of transcriptional activity, and ChIP assays suggest that both FoxO1 and FoxO3a increase the transcription of Sox2 in GBM-SCs. In p53-proficient GBM-SCs, γIR + dual PI3K/mTOR inhibitor treatment reduces stem and progenitor marker expression after a few days while increasing differentiation marker expression, which is associated with impaired self-renewal (sphere formation) and slightly increased cell death. In p53-deficient GBM-SCs, neither FoxO proteins nor stem and progenitor markers or sphere formation decrease upon the combination treatment, and the cells survive. The loss of Sox2 expression in combination-treated, p53-proficient GBM-SCs may be caused by the loss of FoxO proteins, which may be proteolytically degraded by proteasomes upon the p53-mediated induction of Mdm2. Whether Nestin and Musashi are also directly transcriptionally regulated by FoxOs is currently unclear. However, at least the observed Nestin expression changes could also be explained by the known transcriptional regulation of Nestin by Sox2 [61, 62].

Here, we found that p53-deficient GBM-SCs, which are more resistant to combined γIR/PI3K/mTOR inhibition than p53-proficient ones [16], retained stem cell marker expression and self-renewal post-treatment. In contrast, p53-proficient CSCs lost these stemness properties several days post-treatment and differentiated, accompanied by a low amount of cell death (Figure 7); dependence on functional p53 was confirmed by p53 RNA silencing. Loss of stemness in p53-proficient GBM-SCs was accompanied by the loss of FoxO proteins, which so far have been reported to negatively regulate stemness in solid-tumor CSCs [43–47] or related processes [52]. We here provide evidence that, similar to normal-tissue and leukemia stem cells [28, 29, 32, 35, 38, 39, 42], FoxO proteins can have positive effects on stemness in GBM-SCs (Figure 7). Regardless of the p53 functional status, stem and progenitor cell marker expression, particularly that of Sox2, was strongly reduced in FoxO1/3 double (but not single)-deficient GBM-SCs. This suggests that both FoxO proteins can contribute to the control of stem cell marker expression in GBM-SCs. This was confirmed by our finding that both FoxO1 and FoxO3 bind to the *sox2* regulatory regions in GBM-SCs. Functional redundancy between FoxO1 and FoxO3a is also suggested by the low downregulation of stem cell markers in the single FoxO knockdown cells, although the low downregulation in these cells may also be due to the relatively low FoxO knockdown efficacy in these experiments. That FoxO proteins positively regulate stem cell marker expression in the GBM-SCs studied by us was confirmed using a synthetic inhibitor of FoxO1/3 transcriptional activity, and by FoxO3 overexpression which inhibited the loss of stemness proteins upon γIR/PI-103 treatment in p53-proficient GBM-SCs. However, in the absence of FoxO1/3, functional stemness (self-renewal in spheres) and survival upon the combination treatment were strongly reduced only in p53-proficient GBM-SCs. In contrast, in p53-deficient GBM-SCs, FoxO1 and FoxO3 were not required for self-renewal and post-treatment survival, suggesting that these functions can be maintained through the loss of DNA damage-responsive p53 instead (Figure 7).

Our data are in agreement with reports on the role of FoxO proteins in normal-tissue stem cells (which are proficient in p53). FoxO proteins maintain ES cell pluripotency, and FoxO1 controls *sox2* transcription in ES cells [29]. That both FoxO1 and FoxO3 are important for the maintenance of stemness in the brain (as suggested by our data) is also suggested by reports on normal NSCs. FoxO3 has not only been shown to be crucial for the HSC pool but also for self-renewal and proper differentiation of NSCs [35, 36, 38, 39]. Furthermore, it was recently reported that NSCs can only differentiate when they lose FoxO1 expression [40]. Sox2+ NSCs are reduced in FoxO1/3/4 triple-knockout mice [39]. Also the FoxO homologue in *Hydra* seems essential for the indefinite self-renewal of its stem cells [42]. Our data are also in accord with reports that FoxO proteins are essential for stemness of leukemia stem cells [28, 32]. However, our data suggest that in solid tumors (at least in the GBM-SCs studied here) FoxO proteins are mainly required for maintaining functional stemness in p53-proficient CSCs.

Our data are in apparent contrast to previous reports ascribing a negative role to FoxO proteins in stemness regulation in solid-tumor CSCs [43–47]. The discrepancy to reports on stem-like prostate, colon, and breast cancer cells [45–47] may be related to observations that FoxOs can have different functions in different tumor types or depending on the molecular background. Our data suggest that the p53 status is important. The reason for the apparent discrepancy to some of the findings and conclusions by Sunayama *et al.* [43, 53] on FoxO3 in GBM-SCs are currently not clear. Based on experiments with constitutively active FoxO3a, they concluded that FoxO3a promotes differentiation [43]. This discrepancy may, in part, be elucidated by the recent findings of Kim *et al*. [40] who suggested that FoxO expression (in that case FoxO1 in normal NSCs) is crucial both for the maintenance of stemness and at later stages of differentiation. Sunayama *et al.* [53] also reported that a dual PI3K/mTOR inhibitor alone promoted differentiation of GBM-SCs. Although we made similar observations in some GBM-SC lines at higher PI3K/mTOR inhibitor concentrations (data not shown), consistent effects were observed only after combination with γIR and only in p53-proficient GBM-SCs. Thus, the combination treatment with γIR and a dual PI3K/mTOR inhibitor may constitute a pharmacological approach to deplete CSCs in p53-proficient GBMs. This combination treatment can be expected to be more efficient in p53-proficient GBMs than in p53-deficient ones, if such combinations are considered for the clinics.

Our data confirm the notion that loss of functional p53 can contribute to the acquisition of stemness both in malignant disease and during reprogramming of normal differentiated cells [13, 17–19, 54, 55]. They also confirm that DNA damage-responsive p53 can promote differentiation [19, 21]. Moreover, our data suggest a role for DNA damage-responsive p53 (in conjunction with FoxOs) in the control of stemness (Figure 7).

In response to DNA damage, p53 transcriptionally activates FoxO3 [26]. We speculate that the loss of FoxO proteins in p53-proficient CSCs several days after combined γIR/PI3K/mTOR inhibitor treatment is due to proteolytic degradation (Figure 7). FoxO proteins can be degraded by proteasomes after induction of the E3 ligase Mdm2, a transcriptional target of p53 [31, 56]. However, our attempts to prove this assumption by using proteasome inhibitors were not successful because the triple treatment including a proteasome inhibitor was too toxic for the GBM-SCs. Exactly how the FoxO proteins get lost a few days after the combination treatment in p53-proficient CSCs thus remains to be elucidated.

That DNA damage can cause stem cell depletion by triggering differentiation has already been shown for normal-tissue stem cells [57, 58]. Our data and that of others [14, 59, 60] suggest that this may be facilitated by concomitant loss of PI3K pathway activity, which may not only be relevant in p53-proficient tumors but also in normal tissues during aging or genotoxic therapies when sublethal DNA damage and diminished PI3K pathway activity (e.g., upon niche displacement) occur simultaneously. Loss of stemness, associated with differentiation or cell death, upon DNA damage plus/minus PI3K pathway inhibition may serve to remove long-lived stem cells with accumulated DNA damage.

## MATERIALS AND METHODS

### Cell culture and reagents

The GBM-SC lines GBM4, GBM10, GBM22, and GBM36 were established from tumor samples of patients with primary GBM [16, 48]. Informed written consent was obtained before surgery (Approval ID: 349/08). Tumors were dissociated and single cells were cultured under stem cell culture conditions in serum-free Neurobasal Medium (Gibco) supplemented with 20 ng/ml epidermal growth factor/fibroblast growth factor-2 each, B27, non-essential amino acids, penicillin/streptomycin, GlutaMAX, and heparin. The GBM-SC line G166 [49] was purchased from Biorep (Milan, Italy). For expansion, the cells were cultured on plates coated with extracellular matrix (ECM) proteins (mouse sarcoma-derived ECM; Sigma), where they grew as adherent cells. To assess self-renewal, single cells were plated on low-attachment plates (Corning) in stem cell medium at low density. Expression of stem and progenitor markers as well as differentiation markers was assessed by Western blot, as described previously [48]. The cells are tumorigenic upon xenotransplantation in immunodeficient mice (data not shown). The Akt/mTOR inhibitors PI-103 and NVP-BEZ235 were from BioVision and Selleck, respectively. The FoxO1 inhibitor AS1842856 was from Merck Millipore [50].

### γIR

Cells were irradiated using a Gammacell 40 ^137^Cs laboratory irradiator. The dosages used were 2, 5, and 10 Gy.

### Western blot analysis

Cells were lysed in RIPA lysis buffer supplemented with protease inhibitor cocktail (Roche) and the phosphatase inhibitors NaF and Na_3_VO_4_ (Sigma). Cell lysate (40 μg) was separated by SDS-PAGE and blotted onto nitrocellulose. The blots were incubated with the indicated antibodies and developed by enhanced chemiluminescence (GE Healthcare). Antibodies against the following proteins were used: phosphatase and tensin homolog (PTEN), phospho-Akt (Ser473), Akt, phospho-S6 ribosomal protein (Ser235/236), S6 ribosomal protein, FoxO1, FoxO3a, FoxO4, Musashi, p21, GFAP, βIII-tubulin, cleaved caspase 3 (Cell Signaling); actin, Mdm2 (Santa Cruz); p53, Sox2 (R&D Systems); Nestin (Millipore). HRP-conjugated secondary antibodies were purchased from Dianova. Quantification of signals was performed using Image Quant TL (Amersham Bioscience).

### Flow-cytometric detection of cell death

Cells were treated with PI3K/Akt inhibitors for 1 h and then irradiated. At the time points indicated, cells were harvested and those expressing high levels of GFP or RFP were stained only with annexin V; non-transduced cells were stained with annexin V and propidium iodide (PI; Miltenyi Biotec). In addition, FSC/SSC parameters were analyzed to confirm the percentages of apoptotic cells. Cell analyses were performed on a FACSVerse flow cytometer (BD Biosciences).

### Microscopic analysis

Cells were analyzed on an Olympus BX41 fluorescence microscope equipped with a digital camera CC-12 soft imaging system (U-CMAD3, Olympus).

### Sphere-forming assay

Single cells were seeded in low-attachment plates (Corning) and photographs were taken 3 days later.

### Knockdown of p53 and FoxO proteins

Cells were transduced according to the manufacturer's instructions using the following lentiviral particles: human p53 shRNA (GFP-puromycin) and negative-control shRNA (GFP-puromycin) (Amsbio); human FoxO1 shRNA (GFP-puromycin) and non-targeting shRNA (GFP-puromycin) (Thermo Scientific); human FoxO3 shRNA and negative-control shRNA (GFP-hygromycin) (ATCGbio).

### Overexpression of FoxO3

Cells were transduced according to the manufacturer's instructions using the following lentiviral particles: human FoxO3 (RFP-blasticidin) and control (RFP-blasticidin) from Amsbio.

### ChIP assay

The FoxO binding sites in the *sox2* regulatory region have been identified elsewhere [29]. The ChIP assay was performed using a kit (Millipore) according to the manufacturer's instructions. Briefly, after crosslinking with 1% formaldehyde, 2 × 10^6^ cells per sample were lysed and sonicated using a Sonorex Super RK 102H sonicator (Bandelin Electronic) in an ice-water bath for 10 min. Precleared samples were incubated overnight at 4°C with 5 μg anti-FoxO1 (N-18) or 5 μg anti-FoxO3 (N-15) or 5 μg normal goat IgG antibody (all from Santa Cruz). A positive control (anti-RNA polymerase II) and a negative control (normal mouse IgG) were used as indicated in the manufacturer's instructions. Co-immunoprecipitated DNA was reverse crosslinked and analyzed by PCR for binding to the *sox2* promoter. The primer sequences were taken from [29] and were as follows: forward CTTTGTTTGACTCCGTGTAGCGACA; reverse CTCTCCCATTGTCCCGACGTAAAG (fragment size, 126 bp).

### Statistical analysis

Data are presented as mean ± SD and were analyzed by Student's *t*-test, two-tailed, with unequal variance. *P* < 0.05 was considered significant.

## SUPPLEMENTARY MATERIALS


