# Supplementary material for: FoxO proteins or loss of functional p53 maintain stemness of glioblastoma stem cells and survival after ionizing radiation plus PI3K/mTOR inhibition
Source: Oncotarget. 2016 Jul 19;7(34):54883–96. doi: 10.18632/oncotarget.10702 (PMC5342388; doi:10.18632/oncotarget.10702)
Supplement: Supplementary file 1 [file oncotarget-07-54883-s001.pdf]

FoxO proteins or loss of functional p53 maintain stemness of glioblastoma stem cells and survival after ionizing radiation plus PI3K/mTOR inhibition

Supplementary Materials

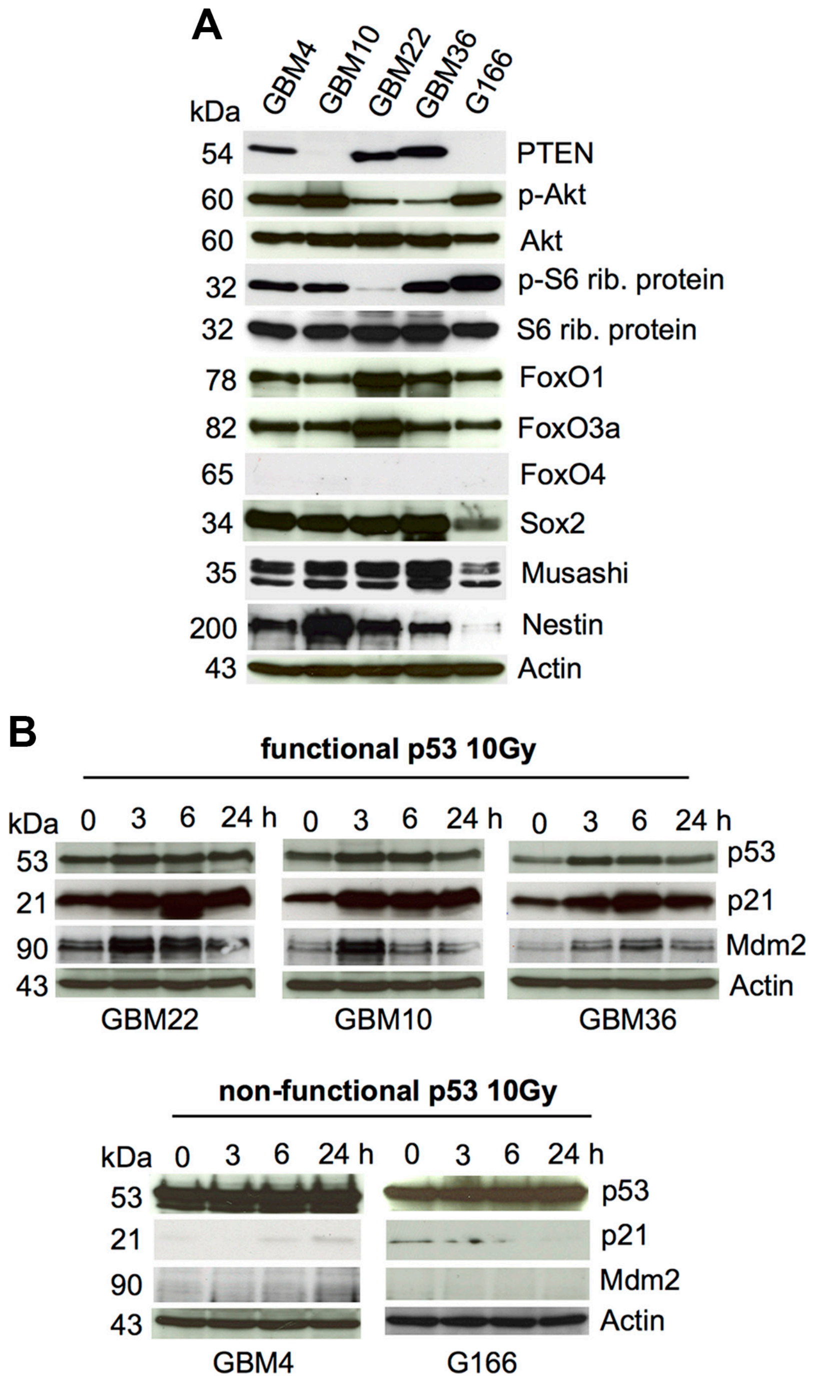

Supplementary Figure S1: (**A**) Heterogeneous expression of components of the PI3K/Akt/mTOR pathway, FoxO proteins, and stemness markers in the GBM-SC lines used in this study. (**B**) Functionality of p53 assessed by Western blot analysis of p53, p21, and Mdm2 after 10-Gy γIR. In each panel, 1 of 2 Western blots with similar results is shown.

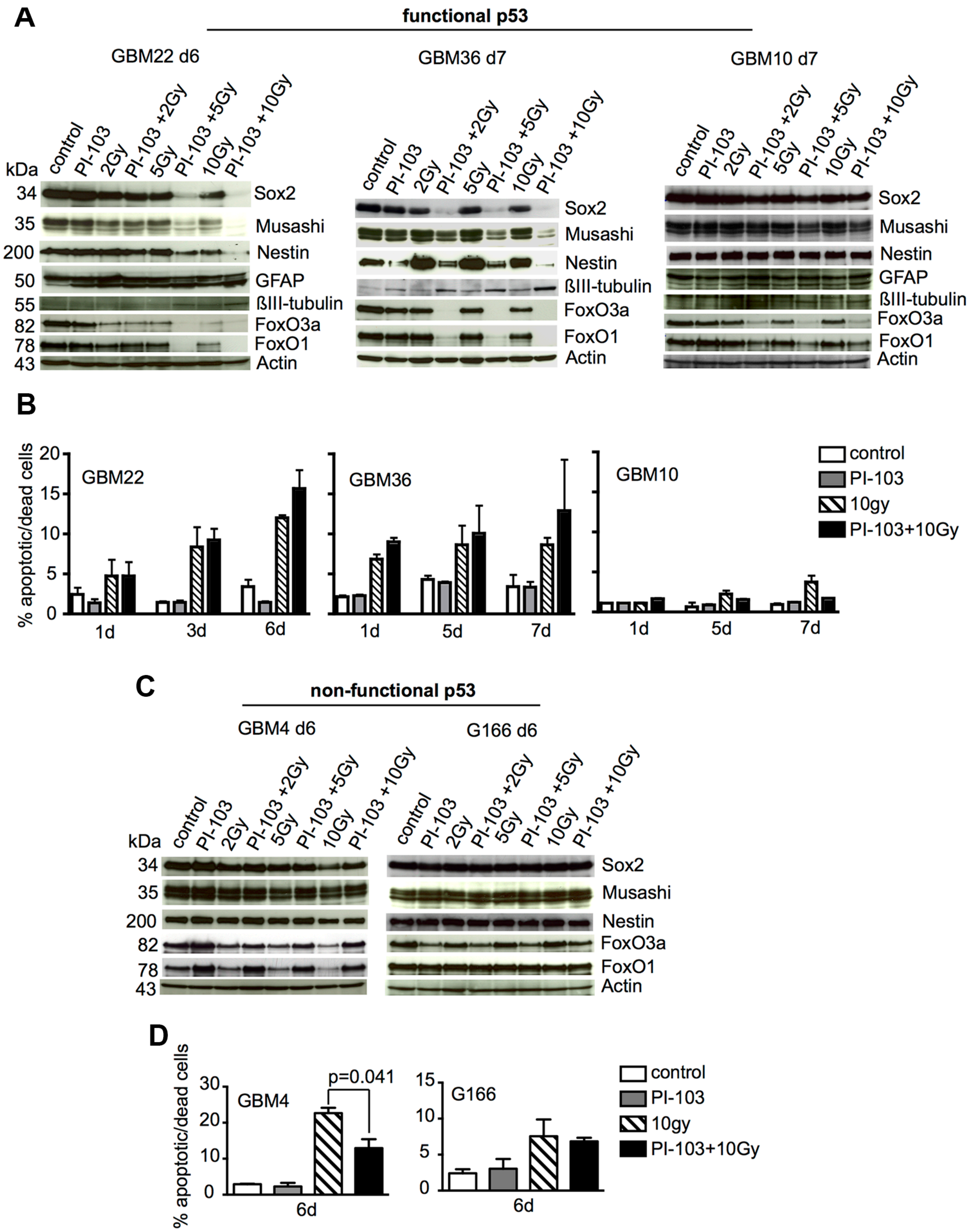

Supplementary Figure S2: Loss of stem cell markers and FoxO proteins in p53-proficient GBM-SCs 6 or 7 days after γIR/PI-103 combination treatment. GBM-SCs with functional p53 (**A** and **B**) or non-functional p53 (**C** and **D**) were incubated with 0.5 µM PI-103 for 1 h, irradiated and analyzed by Western blot for protein expression (A and C) or by flow cytometry to assess the apoptotic/dead cells after incubation with annexin V/PI (B and D). Data in (B) and (D) represent means ± SD from 3 independent experiments.

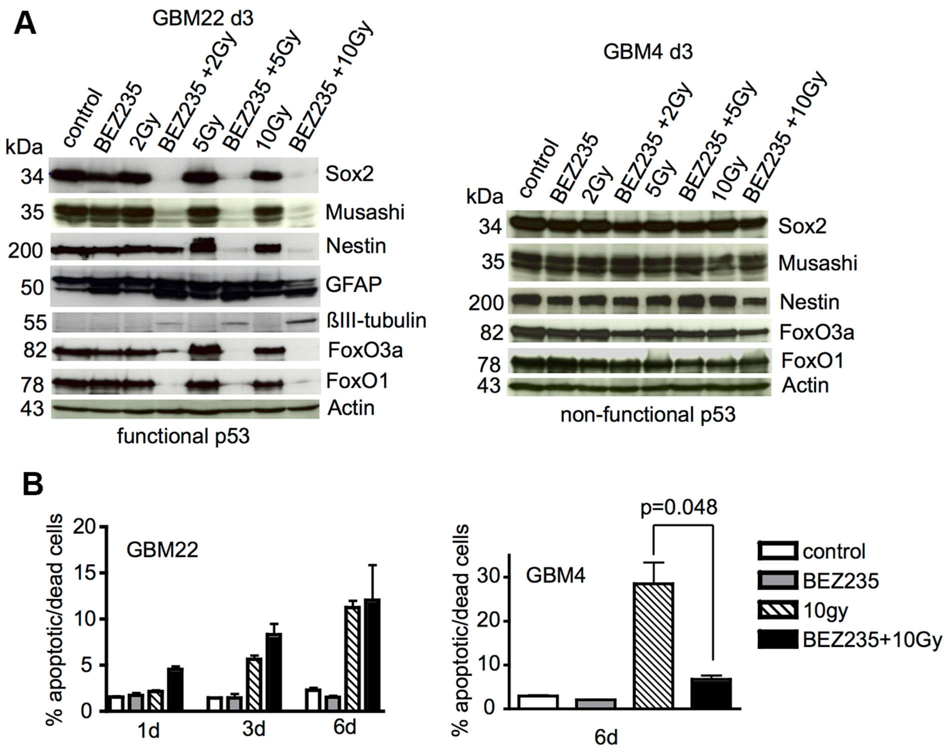

Supplementary Figure S3: Combination treatment with γIR and NVP-BEZ235 causes loss of stemness markers and FoxO proteins in p53-proficient GBM-SCs. GBM-SCs were incubated with 0.5 µM NVP-BEZ235 for 1 h, irradiated and (**A**) analyzed by Western blot for the expression of stemness, differentiation, and FoxO proteins 3 days later (1 of 2 Western blot experiments is shown, each with similar results) and (**B**) analyzed for apoptotic/dead cells by flow cytometry after incubation with annexin V/PI at the time points indicated. Data in (B) represent means ± SD from 3 independent experiments.

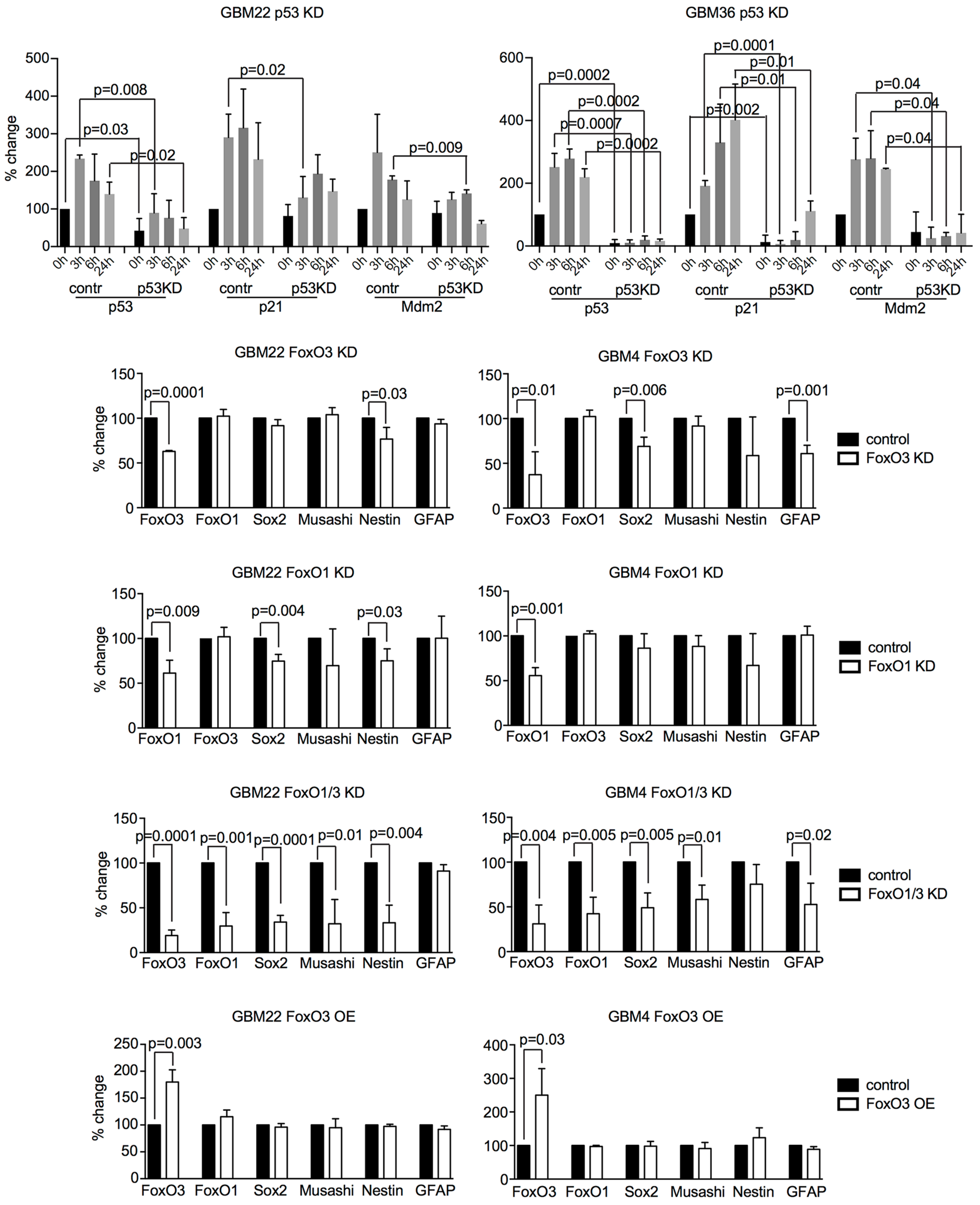

Supplementary Figure S4: Densitometric and statistical analyses of Western blot experiments. Analyses of experiments for which representative blots are shown in Figure 2A (analysis: top row), Figure 3A, 3D (analysis: second row), Figure 4A, 4D (analysis: third row), Figure 5A, 5F (analysis: fourth row), and Figure 6A, 6E (analysis: bottom row). Western blots were quantified using Image Quant TL software and statistically analyzed with Prism software. The results are expressed as means ± SD from at least 3 independent experiments.

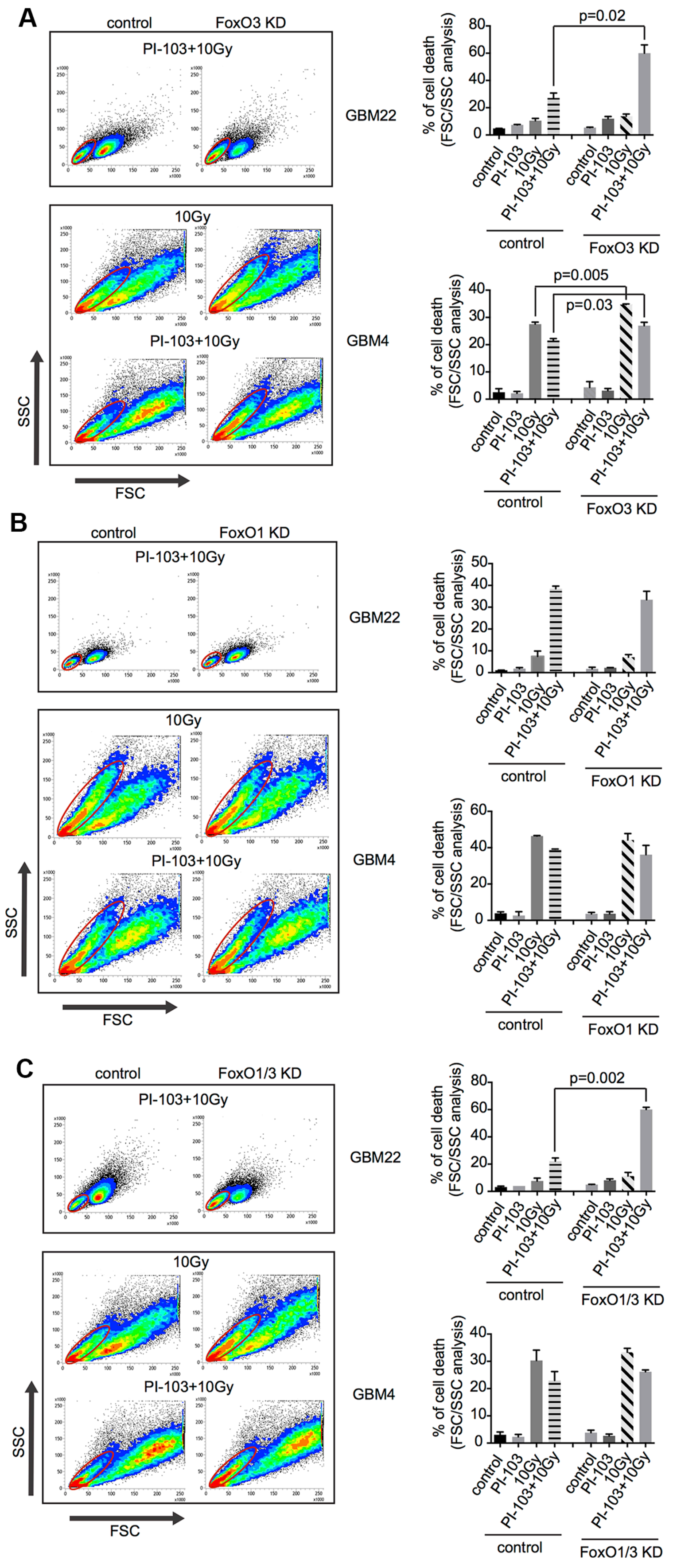

Supplementary Figure S5: Determination of apoptosis by FSC/SSC analysis. (A–C) Confirmation of the annexin V apoptosis assays shown in panels (C) and (F) of Figures 3 and 4 and panels (C) and (H) of Figure 5 by FSC/SSC analysis. Apoptotic cells are circled in the FSC/SSC plots. Data represent means ± SD from 3 independent experiments.

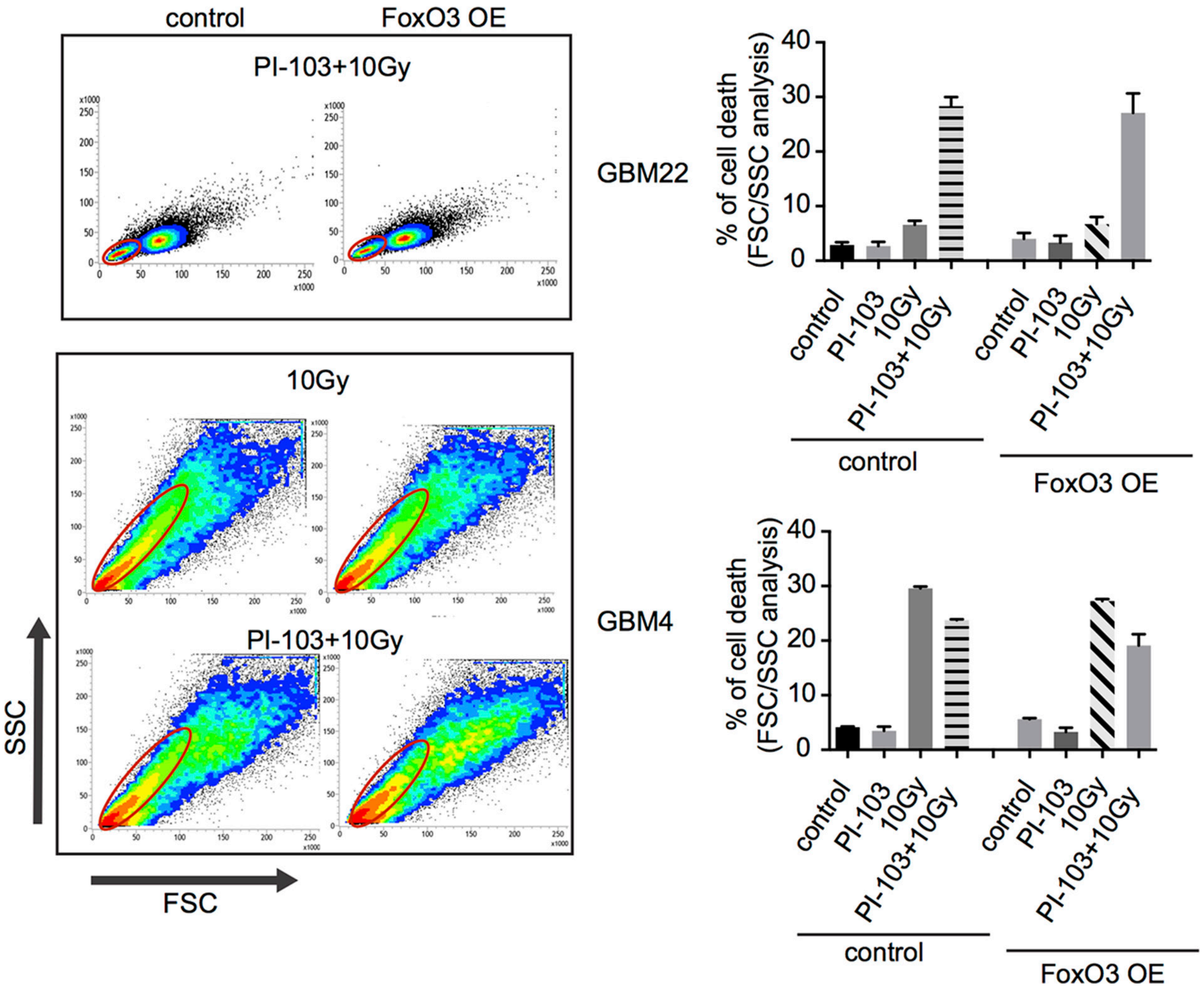

Supplementary Figure S6: Determination of apoptosis by FSC/SSC analysis. Confirmation of the annexin V apoptosis assays shown in panels (C) and (G) of Figure 6 by FSC/SSC analysis. Apoptotic cells are circled in the FSC/SSC plots. Data represent means ± SD from 3 independent experiments.
